# Estimating possible bumblebee range shifts in response to climate and land cover changes

**DOI:** 10.1038/s41598-020-76164-5

**Published:** 2020-11-12

**Authors:** Yukari Suzuki-Ohno, Jun Yokoyama, Tohru Nakashizuka, Masakado Kawata

**Affiliations:** 1 Graduate School of Life Sciences, Tohoku University, 6-3 Aoba, Aramaki-aza, Aoba-ku, Sendai, Miyagi 980-8578 Japan; 2 Faculty of Science, Yamagata University, 1-4-12 Kojirakawa, Yamagata-shi, Yamagata, 990-8560 Japan; 3 Research Institute for Humanity and Nature, 457-4 Motoyama, Kamigamo, Kita-ku, Kyoto, 603-8047 Japan; 4 Present Address: Forestry and Forest Products Research Institute, 1 Matsunosato, Tsukuba, Ibaraki 305-8687 Japan

**Keywords:** Ecological modelling, Conservation biology, Climate-change ecology

## Abstract

Wild bee decline has been reported worldwide. Some bumblebee species (*Bombus* spp.) have declined in Europe and North America, and their ranges have shrunk due to climate and land cover changes. In countries with limited historical and current occurrence data, it is often difficult to investigate bumblebee range shifts. Here we estimated the past/present distributions of six major bumblebee species in Japan with species distribution modeling using current occurrence data and past/present climate and land cover data. The differences identified between estimated past and present distributions indicate possible range shifts. The estimated ranges of *B. diversus*, *B. hypocrita*, *B. ignitus*, *B. honshuensis*, and *B. beaticola* shrank over the past 26 years, but that of *B. ardens* expanded. The lower altitudinal limits of the estimated ranges became higher as temperature increased. When focusing on the effects of land cover change, the estimated range of *B. diversus* slightly shrank due to an increase in forest area. Such increase in forest area may result from the abandonment of agricultural lands and the extension of the rotation time of planted coniferous forests and secondary forests. Managing old planted coniferous forests and secondary forests will be key to bumblebee conservation for adaptation to climate change.

## Introduction

Pollination services are essential ecosystem services that support human life. An estimated 5–8% of global crop production depends on pollination services, equating to an estimated economic value of 235–577 billion U.S. dollars^1^. Among pollinators, the western honey bee *Apis mellifera* is frequently focused on because farmers use managed honey bees for pollination of crops. In 2009, the economic value of pollination of pollinator-dependent crops (apples, almonds, cherries, etc.) in the U.S. was 15 billion U.S. dollars, of which 77% was provided by *A. mellifera*^2^. However, wild non-*Apis* pollinators also play important roles in crop pollination^3^, particularly in countries where the use of *A. mellifera* for pollination of crops grown in open fields is uncommon. For example, in Japan, the economic value of pollination in agricultural production in open fields in 2013 was 360 billion Japanese yen (about 3.7 billion U.S. dollars in 2013), of which only 7.6% was provided by managed *A. mellifera*^4^. There is a great need to conserve wild bees, especially in countries where pollination of crops in open fields depends mainly on wild bees.

Wild bees that provide pollination services have declined worldwide in recent decades^5–10^. The factors that reduce wild bee populations are land cover change, climate change, pesticide use, alien species invasion, pathogen infection, and their combinations^7,11^. Bumblebees (*Bombus* spp.) are wild bees that are effective pollinators in subarctic and temperate zones, and some of their species have declined in Europe and North America^12–16^. The decline of bumblebees in Europe is caused by changes in land cover that result in the degradation of habitat and flower resources^17^. The geographic ranges of European and North American bumblebees have been reduced by climate change^18,19^, and the lower altitudinal limit of *B. alpinus* in the Alps has moved up by climate change^20^. In addition, the decline of bumblebees is caused by pesticides^21,22^, invasion of alien species such as *Bombus terrestris*^23^, and pathogens such as *Nosema* spp^24^.

Investigating bumblebee range shifts and the effects of environmental factors on these range shifts usually requires not only sufficient but also fine spatial-resolution historical and current bumblebee distribution data and environmental data. For countries with a limited amount of historical and current bumblebee distribution data, including Japan, it is difficult to estimate bumblebee range shifts. Therefore, we collected bumblebee occurrence data from photographs taken by citizens in our citizen science monitoring project “*Hanamaru-Maruhana* national census” (bumblebee national census in English), beginning in 2013^25^. Geotagged photographs allowed us to obtain sufficient occurrence data of a fine spatial resolution (n = 70–563, and spatial error < 500 m) for six major bumblebee species in Japan, *Bombus diversus* Smith 1869, *B. ardens* Smith 1879, *B. hypocrita* Perez 1905, *B. ignitus* Smith 1869, *B. honshuensis* Tkalcu 1968, and *B. beaticola* Tkalcu 1968. Some previous studies have reported a low accuracy of species identification by citizens in citizen science monitoring^26,27^, but in our project, one of the authors (J. Yokoyama, who is an expert in bumblebee identification) identified bumblebee species in all photographs. We were able to identify bumblebee species using photographs because major bumblebee species in Japan are easier for experts to distinguish based on the body characteristics than those of Europe and North America^25^ (see https://hanamaruproject.s1009.xrea.com/hanamaru_project/identification_E.html for the details of their body color and shape).

When historical occurrence data is limited, it is necessary to estimate past species distributions using models. We focused on species distribution models built using current occurrence data and present environmental data because they allow us to estimate past distributions even in the absence of historical occurrence data. Species distribution models can mitigate the negative effect of the spatial bias of occurrence data through background manipulation^28^, and estimate past/present distributions using current occurrence data and past/present environmental data^29^. Estimated past and present distributions were compared to identify areas where distributions may have changed. If the accuracy of the estimated distributions is high, these areas correspond to possible range shifts, assuming that bumblebees follow climate and land cover changes closely and that their distributions have reached an equilibrium.

**Figure 1:**
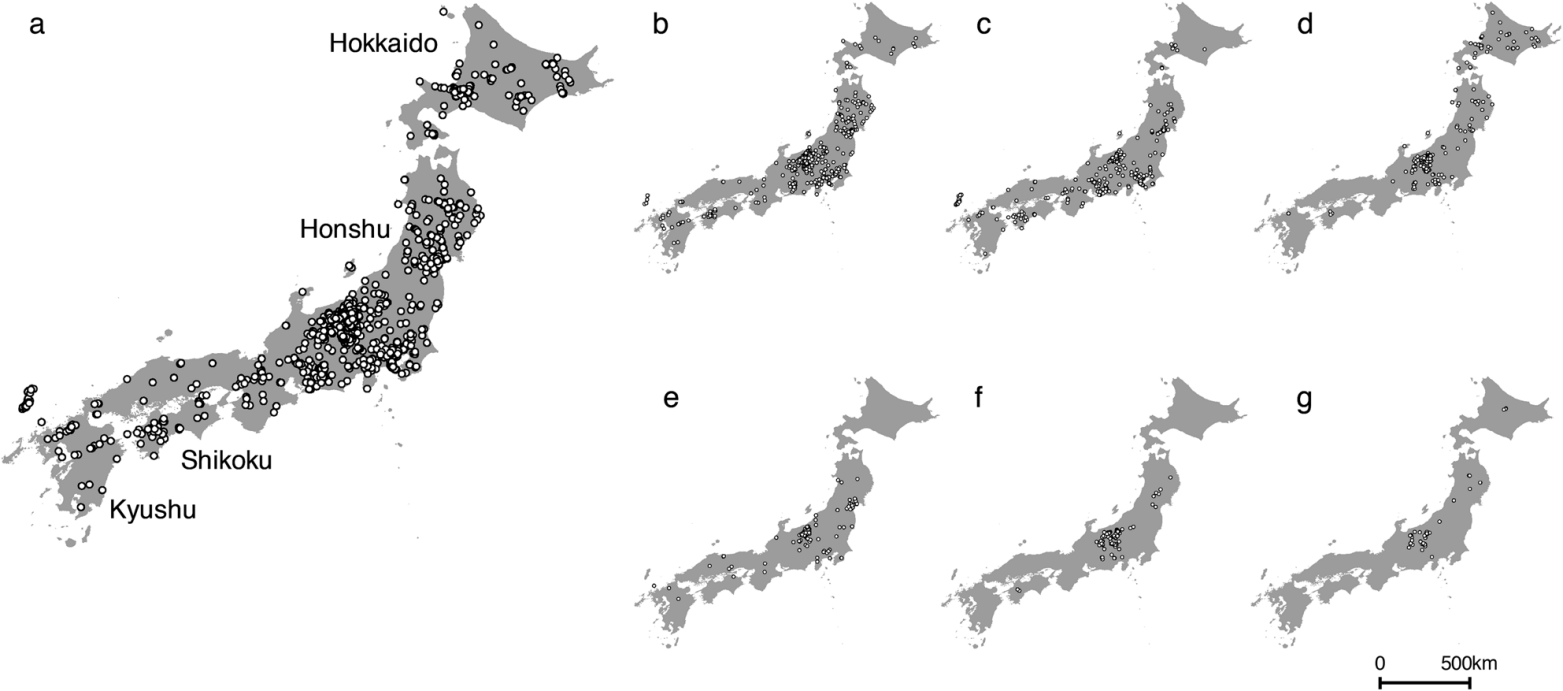
Bumblebee occurrence data used to estimate past/present distributions in Maxent (after Suzuki-Ohno et al.^25^). (**a**) 15 bumblebee species (required to make a bias file used in Maxent), (**b**) *Bombus diversus*, (**c**) *B. ardens*, (**d**) *B. hypocrita*, (**e**) *B. ignitus*, (**f**) *B. honshuensis*, and (**g**) *B. beaticola*. (**a**) was required to make a bias file used in Maxent. This map was drawn with the software ArcGIS ver. 10.0 (https://www.arcgis.com/features/index.html).

In this study, we aimed to estimate possible bumblebee range shifts in Japan from 1987 to 2013. We estimated past/present bumblebee distributions using current bumblebee occurrence data (Fig. 1) and past/present climate and land cover data (Table 1) with Maxent^30,31^. We compared the estimated past and present distributions of each species and identified differences between the two as possible range shifts. In addition, to focus on the effects of land cover change, we estimated past/present bumblebee distributions considering only land cover change by using the average climate data instead of the past/present climate data in our analysis. We compared the estimated past and present distributions, and investigated land cover changes in the areas of estimated range reduction. Based on these estimates, detailed further investigations can be conducted by scientists into the areas of possible range shifts. The identification of areas of possible range shift may be valuable for enhancing researches on further range shifts and bumblebee conservation. In addition, these estimates will be useful for identifying problems that bumblebees face and devising possible conservation actions^32^.

**Table 1:** Environmental variables used in Maxent.

Environmental data (year)	Variables (unit)
Climate data	Annual total precipitation (mm)
(Present/past: 1978–1987/2004–2013)	Annual mean temperature (°)
(30-year average: 1981–2010)	Annual mean solar radiation (MJ m^−2^)
Land cover data (1987, 2014)	Forest area (m^2^)
	Paddy field area (m^2^)
	Other agricultural area (agricultural land other than paddy field) (m^2^)
	Wasteland area (m^2^)
	Other land area (artificial open space with neither woods nor buildings) (m^2^)
	Building area (m^2^)
	River and lake area (m^2^)
	Beach area (m^2^)
	Sea area (m^2^)

## Results

### Estimating possible bumblebee range shifts in response to climate and land cover changes

#### Accuracy of estimated distributions

Present distributions were estimated using present climate and land cover data (hereafter referred to as presD-CL). Area under the receiver operating characteristic curve (AUC) values were used to evaluate the accuracy of the presD-CL. The mean AUC values of the presD-CL were high for all six studied species: 0.764 for *B. diversus*, 0.820 for *B. ardens*, 0.835 for *B. hypocrita*, 0.890 for *B. ignitus*, 0.932 for *B. honshuensis*, and 0.974 for *B. beaticola*. Then, past distributions were estimated using past climate and land cover data (hereafter referred to as pastD-CL). The mean percentages that the pastD-CL included test data of past distributions (collection locations from specimen data from the 1980s, n = 14–60) were checked to evaluate the accuracy of the pastD-CL. The pastD-CL thresholded by minimum training presence (MTP) included almost all of the test data of past distributions (*B. diversus*: 99.2%*, B. ardens*: 99.7%, *B. hypocrita*: 99.7%, *B. ignitus*: 100%, *B. honshuensis*: 87.1%, and *B. beaticola*: 85.7%).

#### Evaluation of possible range shifts

**Figure 2:**
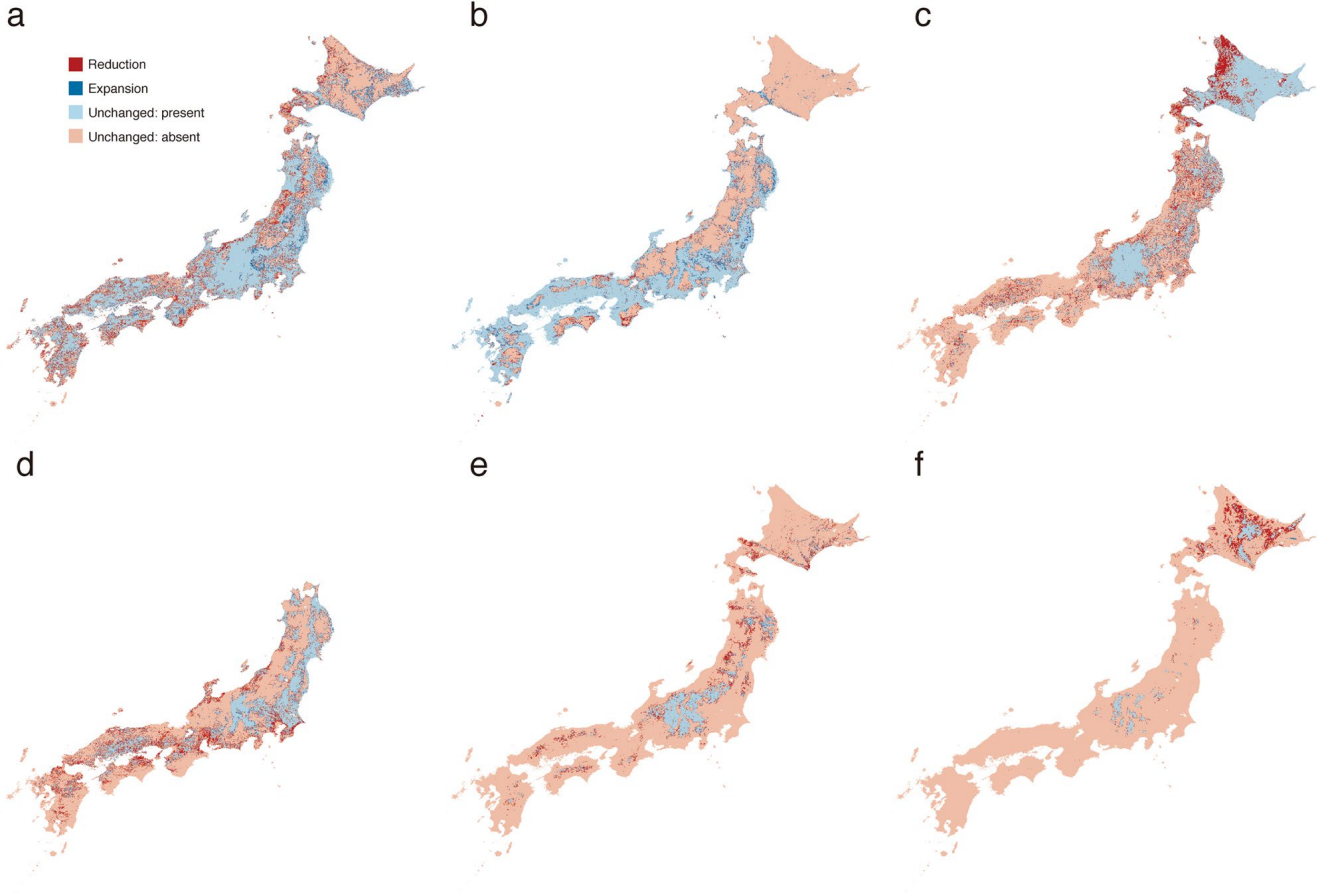
Possible range shifts based on differences between presD-CL and pastD-CL evaluated based on equate entropy of thresholded and original distribution (EETO). presD-CL is estimated present distribution using present climate and land cover data, and pastD-CL is estimated past distribution using past climate and land cover data. EETO is an intermediate threshold between conservative and challenging thresholds in Maxent. (**a**) *Bombus diversus*, (**b**) *B. ardens*, (**c**) *B. hypocrita*, (**d**) *B. ignitus*, (**e**) *B. honshuensis*, and (**f**) *B. beaticola*. Blue and red represent estimated range reduction and expansion, respectively. Sky blue indicates no difference in estimated present areas, whereas pink indicates no difference in estimated absent areas. This map was drawn with the software QGIS ver. 2.14 (https://qgis.org/en/site/).

Comparison of the pastD-CL and presD-CL suggested that changes in climate and land cover shrank the estimated ranges of *B. diversus*, *B. hypocrita*, *B. ignitus*, *B. honshuensis*, and *B. beaticola*, but expanded the estimated range of *B. ardens* (Table 2). These estimated changes were consistent in all analyses based on six thresholds (MTP, 10 percentile training presence (10PTP), equal training sensitivity and specificity (ETSS), maximum training sensitivity plus specificity (MTSS), balance training omission, predicted area and threshold value (BTPT), and equate entropy of thesholded and original distribution (EETO) in Table 2). The estimated range reductions were biased to the lower altitudinal limits of estimated past ranges. Figure 2 shows possible range shifts based on EETO, which is an intermediate threshold between conservative and challenging thresholds. In other words, areas thresholded by EETO were of intermediate size relative to areas thresholded by the other five thresholds. The estimated *B. diversus* and *B. honshuensis* range reductions were observed in various regions of the Japanese archipelago (Fig. 2a,e). The estimated *B. hypocrita* and *B. beaticola* range reductions were severe in the northern island, Hokkaido (Fig. 2c,f). The estimated *B. ignitus* range reduction was frequently observed near coastlines (Fig. 2d). On the other hand, the estimated range of *B. ardens* expanded beyond the upper altitudinal limit of the estimated past range of this species (Fig. 2b).

**Table 2:** Estimated range reduction and expansion based on differences between presD-CL and pastD-CL evaluated based on six thresholds. presD-CL is estimated present distribution using present climate and land cover data, and pastD-CL is estimated past distributions using past climate and land cover data. Mean of the area (M) and the percentage of difference (Δ) were calculated using 10 replicates. The area is the number of presence cells (1 km^2^) in pastD-CL.

	*B. diversus*	*B. ardens*	*B. hypocrita*	*B. ignitus*	*B. honshuensis*	*B. beaticola*
**Minimum training presence (MTP)**						
M	353,035.6	353,290.4	363,308.4	268,256.1	154,213.4	82,626.7
Δ	− 0.0118%	1.54%	− 2.02%	− 2.08%	− 13.0%	− 32.2%
**10 percentile training presence (10PTP)**						
M	214,795.7	155,386.5	184,868.7	108,410.9	36,888.6	22,279.7
Δ	− 9.57%	9.41%	− 21.0%	− 25.5%	− 21.5%	− 49.6%
**Equal training sensitivity and specificity (ETSS)**						
M	99,943.7	83,415	85,002.4	77,324	27,838.5	30,292.8
Δ	− 13.8%	20.9%	− 35.4%	− 29.4%	− 19.8%	− 50.2%
**Maximum training sensitivity plus specificity (MTSS)**						
M	95,652.7	98,874.2	62,476.8	90,583.2	28,373.4	36,623.1
Δ	− 13.9%	17.3%	− 30.7%	− 27.6%	− 19.7%	− 48.8%
**Balance training omission, predicted area and threshold value (BTPT)**						
M	347,251.4	280,877.9	267,114.4	186,582.1	149,940.7	88,380.8
Δ	− 0.425%	3.49%	− 12.7%	− 14.9%	− 13.4%	− 29.7%
**Equate entropy of thresholded and original distribution (EETO)**						
M	232,786.7	166,065.7	168,237	111,922.9	52,770.1	35,245
Δ	− 8.45%	8.72%	− 23.8%	− 24.9%	− 24.1%	− 49.5%

#### Environmental changes

The percent contribution and permutation importance of environmental factors were used to estimate changes in environmental factors that led to the estimated range changes. According to the percent contribution and the permutation importance in Maxent, mean temperature, mean solar radiation, and forest area are important environmental factors for the estimation of bumblebee distributions (Table S1 in Appendix S1 in Supplementary information). Both the percent contribution and the permutation importance of mean temperature were high for all six species, especially for the alpine species *B. beaticola* (percent contribution: 23.3% for *B. diversus*, 23.3% for *B. ardens*, 33.9% for *B. hypocrita*, 18.0% for *B. ignitus*, 39.8% for *B. honshuensis*, and 60.9% for *B. beaticola*, permutation importance: 23.9% for *B. diversus*, 22.9% for *B. ardens*, 47.5% for *B. hypocrita*, 40.4% for *B. ignitus*, 26.2% for *B. honshuensis*, and 86.0% for *B. beaticola*). Since the mean temperature in 2004–2013 increased by 0.89 °C from 1978–1987 (see Environmental data section in Methods), increasing temperature was the major factor involved in shifting estimated bumblebee ranges. The estimated range of *B. ardens* was expanded because relatively high temperatures are suitable for this species (Fig. S1b in Appendix S1 in Supplementary information). Although the percent contribution of mean solar radiation was high for five species (Table S1 in Appendix S1 in Supplementary information), changes in solar radiation did not correspond closely to the areas of estimated range reduction. The percent contribution of forest area was also high for four species, but large changes in forest area occurred in small local regions.

### Estimating possible bumblebee range shifts in response to land cover change alone

#### Accuracy of estimated distributions

AUC values in Maxent were used to evaluate the accuracy of the estimated present distributions using average climate and present land cover data (hereafter referred to as presD-L). The mean AUC values of presD-L were high for all six species: 0.756 for *B. diversus*, 0.817 for *B. ardens*, 0.822 for *B. hypocrita*, 0.883 for *B. ignitus*, 0.936 for *B. honshuensis*, and 0.973 for *B. beaticola*. Then, past distributions were estimated using past land cover data (hereafter referred to as pastD-L). The mean percentages that the pastD-L included test data of past distributions (collection locations from specimen data from the 1980s, n = 14–60) were checked to evaluate the accuracy of the pastD-L. The pastD-L thresholded by MTP included most of the test data of past distributions (*B. diversus*: 98.2%*, B. ardens*: 99.7%, *B. hypocrita*: 99.0%, *B. ignitus*: 100%, *B. honshuensis*: 83.3%, and *B. beaticola*: 78.6%).

#### Evaluation of possible range shifts

**Figure 3:**
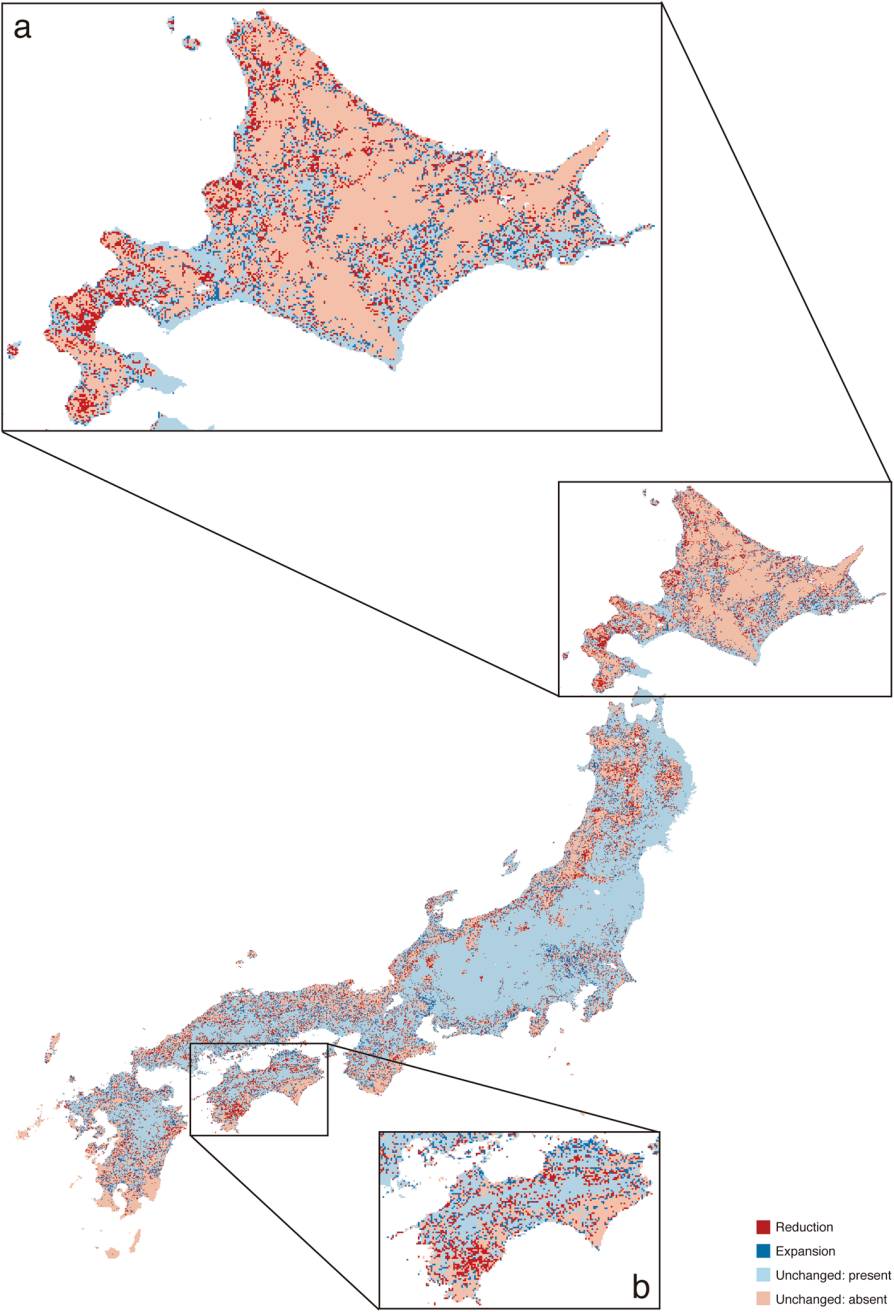
Possible *Bombus diversus* range shift based on differences between presD-L and pastD-L evaluated based on equate entropy of thresholded and original distribution (EETO). presD-L is estimated present distribution using average climate data and present land cover data, and pastD-L is estimated past distribution using average climate data and past land cover data. EETO is an intermediate threshold between conservative and challenging thresholds in Maxent. Red and blue represent estimated range reduction and expansion, respectively. Sky blue indicates no difference in present areas, whereas pink indicates no difference in absent areas. (**a**) Hokkaido and (**b**) Shikoku. This map was drawn with the software QGIS ver. 2.14 (https://qgis.org/en/site/).

Comparison of presD-L and pastD-L showed that land cover change shrank the estimated range of *B. diversus* but expanded those of the other five species (Table 3). These estimated changes were consistent in all analyses using six thresholds (MTP, 10PTP, ETSS, MTSS, BTPT, and EETO in Table 3). The percentage of estimated *B. diversus* range reduction was low because the estimated range reduction in response to land cover change occurred in local regions. Figure 3 shows possible *B. diversus* range shifts based on an intermediate threshold, EETO. The estimated *B. diversus* range reduction occurred frequently in the western regions of Hokkaido (Fig. 3a) and Shikoku (Fig. 3b) according to analysis based on EETO. The estimated range expansion of the other five species occurred in small areas of various regions irrespective of latitude.

**Table 3:** Estimated range reduction and expansion based on differences between presD-L and pastD-L evaluated based on six thresholds. presD-L is estimated present distribution using average climate data and present land cover data, and pastD-L is estimated past distribution using average climate data and past land cover data. Mean of the area (M) and the percentage of difference (Δ) were calculated using 10 replicates. The area is the number of presence cells (1 km^2^) in pastD-L.

	*B. diversus*	*B. ardens*	*B. hypocrita*	*B. ignitus*	*B. honshuensis*	*B. beaticola*
**Minimum training presence (MTP)**						
M	360,567.8	350,768.2	341,197.8	273,290.1	117,315	54,568.2
Δ	− 0.365%	1.14%	0.924%	0.551%	6.03%	1.47%
**10 percentile training presence (10PTP)**						
M	192,053.6	170,564.9	125,860	87,775.6	22,206.5	13,082.4
Δ	− 3.38%	4.73%	0.971%	4.35%	6.68%	5.13%
**Equal training sensitivity and specificity (ETSS)**						
M	85,870.7	91,713.2	58,770.3	53,167.7	20,659.8	16,688.3
Δ	− 0.754%	11.3%	4.09%	7.07%	6.44%	4.54%
**Maximum training sensitivity plus specificity (MTSS)**						
M	94,149.7	106,783.9	64,916.6	50,873.3	25,319.7	21,899.9
Δ	− 1.19%	9.22%	3.61%	7.38%	7.16%	4.08%
**Balance training omission, predicted area and threshold value (BTPT)**						
M	340,052.7	263,560.4	233,491.3	157,102.4	117,315	62,099.6
Δ	− 0.899%	2.59%	2.05%	1.69%	6.03%	0.978%
**Equate entropy of thresholded and original distribution (EETO)**						
M	229,879.7	173,350.1	141,685.7	90,857.7	38,965.9	19,011
Δ	− 2.86%	4.68%	1.37%	4.17%	7.19%	4.27%

#### Environmental changes

**Figure 4:**
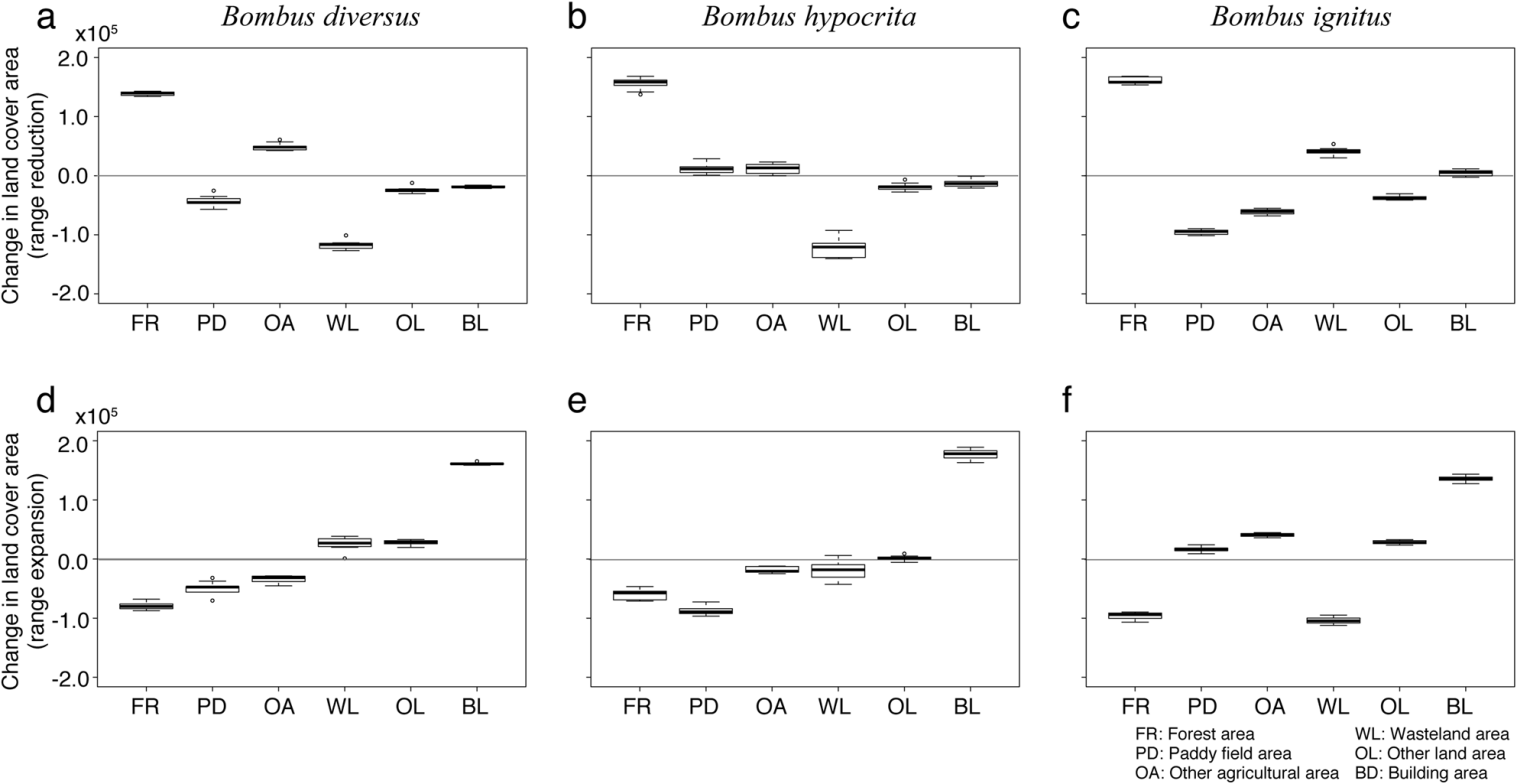
Turkey boxplot of changes in land cover area in areas of estimated range reduction or expansion for *Bombus diversus*, *B. hypocrita*, and *B. ignitus* evaluated based on equate entropy of thresholded and original distribution (EETO). EETO is an intermediate threshold between conservative and challenging thresholds in Maxent. Boxplot shows the mean change of each estimate for 10 replicates. Uppers: mean change areas in the areas of estimated range reduction. (**a**) *B. diversus*, (**b**) *B. hypocrita*, and (**c**) *B. ignitus*. Lowers: Mean change areas in the areas of estimated range expansion. (**d**) *B. diversus*, (**e**) *B. hypocrita*, and (**f**) *B. ignitus*. *FR* forest area, *PD* paddy field area, *OA* other agricultural area, *WL* wasteland area, *OL* other land area, *BL* building area.

The percent contribution and permutation importance of environmental factors were used to estimate changes in land cover areas that led to the estimated range changes. Among land cover areas (Table S2 in Appendix S2 in Supplementary information), the percent contribution and permutation importance of forest area were high for *B. diversus*, *B. hypocrita*, and *B. ignitus* (percent contribution: 33.9% for *B. diversus*, 18.3% for *B. hypocrita*, 43.1% for *B. ignitus*, permutation importance: 25.7% for *B. diversus*, 12.9% for *B. hypocrita*, 15.0% for *B. ignitus*). The major land cover changes in the areas of estimated range reduction based on EETO was an increase in forest area (Fig. 4a–c). In addition, forest areas were decreased in the areas of estimated range expansion by EETO (Fig. 4d–f). In Japan, forest areas have substantially increased in some local regions, but have slightly changed in most areas over the past 26 years. The estimated past distribution of *B. diversus* was the widest among *B. diversus*, *B. hypocrita*, and *B. ignitus* (M in Table 3), and included local regions where large-scale increases in forest areas happened to occur. The net range reduction of *B. diversus* was attributed to the large-scale increases in forest areas in local regions, such as those in Hokkaido and Shikoku (Fig. 3). The estimated past distributions of *B. hypocrita* and *B. ignitus* did not always include these local regions where such large-scale increases in forest areas happened to occur, and their distributions were estimated to have expanded because forest areas have frequently and slightly decreased within their estimated distributions. The net range expansion of other three species was attributed to the changes of other land cover areas. Since the land cover type with the highest percent contribution for *B. ardens* was building area (44.2%), and that for *B. honshuensis* and *B. beaticola* was other land (artificial open space with neither woods nor buildings) area (9.8% for *B. honshuensis* and 8.8% for *B. beaticola*), increases in these land cover areas lead to slight range expansion for those species (Table S2 and Fig. S4 in Appendix S2 in Supplementary information).

We checked vegetation types in the areas of estimated *B. diversus* range reduction. Since land cover data did not include information on vegetation types, a vegetation map was used to investigate vegetation types in the areas of estimated *B. diversus* range reduction. The vegetation types in these areas differed between regions, but commonly included planted coniferous forests. In Hokkaido (Fig. 3a), the major vegetation type in areas where the estimated range of *B. diversus* was reduced were natural forests. The second major type was agricultural land, and the third major type was planted coniferous forests composed of deciduous conifers such as *Larix kaempferi* (6.9%) and evergreen coniferous species such as *Abies sachalinensis* forest (6.4%). In Shikoku (Fig. 3b), the major vegetation type was planted evergreen coniferous forests, including planted *Cryptomeria japonica*, *Chamaecyparis obtusa*, and *Chamaecyparis pisifera* forests (53.7%). The second major type was secondary forests, which are often observed in agricultural lands.

## Discussion

Our results suggested that the estimated ranges of five bumblebee species, *B. diversus*, *B. hypocrita*, *B. ignitus*, *B. honshuensis*, and *B. beaticola* shrank, but the estimated range of *B. ardens* expanded as a result of climate and land cover changes over the past 26 years (Table 2 and Fig. 2). Increasing temperature caused by global warming was considered to be the major factor responsible for the reduction in the estimated ranges of these five species. The estimated range reductions were biased to the lower altitudinal limits of their estimated past ranges, regardless of latitude. The estimated range reductions based on EETO showed that the estimated *B. hypocrita* and *B. beaticola* range reductions were severe in the northern island of Japan, Hokkaido (Fig. 2c,f). Since *B. hypocrita* favors cold temperatures and inhabits moderate sloping grasslands in Hokkaido, and the typical alpine species *B. beaticola* also favors cold temperatures^33^, we conclude that the estimated range reductions in Hokkaido were severe due to global warming. On the other hand, higher temperatures are suitable for *B. ardens* (Fig. S1b in Appendix S1 in Supplementary information), so the range of this species was estimated to expand due to global warming.

Changes in temperature and precipitation due to global warming have reduced bumblebee ranges in Europe and North America^19^. Temperature and precipitation are important environmental factors affecting bumblebee’s mortality and fecundity^34^, or abundances through changes in floral phenology^35^. In the present study, the percent contribution and permutation importance of mean temperature were high for all six studied species, but those of annual precipitation were relatively low. This could be attributed to the fact that annual precipitation is higher in Japan than in Europe and North America.

Our results also showed that the estimated range of the common species *B. diversus* shrank, but the estimated ranges of the other five studied species expanded due to land cover change (Table 3). The major land cover change in the areas of estimated *B. diversus* range reduction was an increase in forest areas (Fig. 4a). There was no net range reduction of *B. hypocrita* and *B. ignitus* (Table 3), but the major land cover change in the areas of estimated range reductions for these two species was also an increase in forest area (Fig. 4b,c). There is a risk of net *B. hypocrita* and *B. ignitus* range reductions if the large-scale increases in forest areas occur within their ranges.

Generally, an increase in forest area should have a positive effect on wild bees^36–38^, but there are two scenarios in which a large forest area could reduce bumblebee ranges. The first scenario is one in which large areas of planted coniferous forests are abandoned without harvesting. In Japan, two-thirds of the land area is forest, two-fifths of the forest area is plantation, and half of planted coniferous forests are old forests (planted over fifty years ago)^39^. Old planted coniferous forests have few understory flowering plants due to a lack of sunlight on the forest floor^40^. They may not provide suitable habitats for bumblebees, especially those composed of deciduous coniferous species such as *Larix kaempferi* (a domestic alien species in Hokkaido: Fig. 3a), and temperate evergreen coniferous species such as *Cryptomeria japonica* and *Chamaecyparis* spp. (species with limited natural distribution in Shikoku: Fig. 3b). In the second scenario, secondary forests in abandoned agricultural areas expanded beyond the optimum size. In our previous study^25^, estimation results suggested that medium-sized forest areas (0.35–0.7 × 10^6^ m^2^ in 1 km^2^) were suitable for four species, *B. diversus*, *B. ardens*, *B. hypocrita*, and *B. ignitus*. Many bumblebee species require natural or semi-natural grasslands as foraging sites^41^, and some bumblebee species prefer forest edges from which they can easily visit foraging sites outside of the forests^42^. Excessive increases in forest area will reduce grassland foraging sites and habitat suitability for these bumblebees. In Kerr et al.^18^, they concluded that alpine tree line advances reduce bumblebee ranges by decreasing nesting and foraging sites in historically open areas.

Based on our results, further field studies are needed to reveal whether bumblebee ranges shrank in areas with increasing forest area, or whether excessive increases in planted coniferous forests and/or secondary forests decrease habitat suitability for bumblebees. Grasslands and shrubs are preferred by bumblebees in many countries, but the preference for coniferous forests may differ among countries or regions within a country. In Vermont, U.S.A., there is a positive association between evergreen coniferous forest and bumblebee abundance^43^, although evergreen coniferous forest has a relatively low diversity of flowering plants. This result may reflect the presence of nesting and foraging sites in wetlands and at forest edges. It is necessary to focus not only on forest type, but also on forest age and the presence or absence of nesting and foraging sites.

After verification by field studies, we can suggest possible conservation actions for bumblebees. We cannot stop global warming immediately; however, we can act to adapt to climate change with respect to land cover changes. Habitat networks connecting existing habitat to new areas that will become suitable for target species as a result of global warming have been proposed as a strategy to adapt to climate change^44^. An effective way to increase connectivity to higher altitude regions is reducing unfavorable land cover for bumblebees, especially in regions at the upper altitudinal limits of their distributions. In Japan, we could reduce old coniferous forests by promoting the logging and regeneration of coniferous forest plantations. Most planted coniferous forests in Japan have matured without harvesting and regeneration due to the depression of the forestry industry^39,45^. Low timber prices together with decreases in the labor force due to Japan’s aging population and depopulation in rural areas makes it difficult to develop effective infrastructure for yarding or log conveying. A political measure of adequate infrastructure and management is required to promote the logging and regeneration of conifer plantations. When the cost of such measures is high, reconversion from conifer plantations to broad-leaved forests will be effective as an alternative option. Retain favorable land covers for bumblebees may also be effective. Semi-natural grasslands traditionally managed by humans in agricultural areas, such as socio-ecological production landscapes (SEPLs, or Satoyama in Japanese), are important foraging sites not only for bumblebees^46,47^ but also for solitary bees^47^. Annual mowing and/or burning of semi-natural grasslands by humans is necessary for the maintenance of these landscapes because abandoned grasslands become forests through ecological succession. However, by 2050, 21% of SEPLs in Japan are expected to be abandoned due to an aging population and depopulation in rural areas^48^. When semi-natural grasslands traditionally managed by humans are abandoned, the diversity of plants and herbivorous insects in these grasslands declines^49,50^. In Japan, some nonprofit organizations manage semi-natural grasslands and SEPLs using their own methods. In addition, there have been some efforts to restore abandoned agricultural land to flower fields. Financial and scientific support from administrators, scientists, and museum staff is required to enhance these citizen activities. We discussed them as examples in Japan, but similar problems may occur in countries with aging populations or high urban concentration. In all land-use scenarios of the conterminous United States from 2001 to 2051, forest areas were projected to increase but range areas (grasslands and shrublands) were projected to decline^51^.

Our results identify possible range shifts and are useful for devising possible conservation actions for bumblebees, but we must interpret these possible range shifts carefully because species distribution models have limitations. The limitations of the species distribution models are related to (1) data issues, including data quality; (2) modeling techniques, mainly regarding the evaluation of predictive ability; (3) the assumptions of species distribution models; and (4) the interpretation of the results^52^. First, regarding (1) data issues, the present study solved data quality issues (e.g., misidentification of bumblebee species and bias of occurrence data in citizen science monitoring) through expert species identification and a background manipulation in Maxent. Next, regarding (3) modeling techniques, the present study attempted to solve index issues (e.g., AUC) in model performance by using test data of past distributions (specimen data) in addition to AUC values. However, we considered that specimen data used in the present study might not evaluate the accuracy of estimated past distributions because the collection localities of specimens were biased, and frequently included in both estimated past and present distributions (see Appendices S1 and S2 in Supplementary information for more details of evaluation). The remaining major limitations were (3) the assumptions of species distribution models and (4) the interpretation of the results. Species distribution models can predict past/present distributions if all factors limiting the distribution are included in the models, the distribution has reached equilibrium, and the target species responds to changes in the environment by dispersion or local extinction.

We must consider the probability that these assumptions are not satisfied, and interpret our results carefully. Possible range shifts estimated by Maxent may not corresponds to actual range shifts because all factors limiting the distributions are not always included in the model. We were not able to include factors related to pesticides, alien species, and pathogens in the model because there were only two types of agricultural land in the environmental data, and insufficient data on alien species and pathogens. Even if all factors are included in the model, the relationship between environmental factors and bumblebee distributions may change due to evolution. When estimating long-term range shifts, it is necessary to consider the effects of evolution on distributions. In addition, current species distributions are not always at equilibrium^53^. Bumblebee distributions may not strongly reflect temperature changes because of low dispersal rates^54^. If the current occurrence data used in the present study represented bumblebee distributions that were not at equilibrium due to low dispersal rates, the actual past distributions and range reductions would be different from our estimates. If low dispersal rates resulted in local extinction at unsuitable sites without colonization of new sites, the current occurrence data would show a narrow range of suitable environments. In this case, estimated distributions and range reductions would be underestimates because the past distributions were estimated using narrower environmental ranges. If low dispersal rates resulted in only reduced abundances at unsuitable sites^55^, the current occurrence data would show environmental ranges that do not reflect actual suitable environments. In this case, estimated past distributions and range reductions would be in different locations from actual distributions and range reductions. To account for the possibility of different range shifts, we must investigate both the distribution and abundance of bumblebees carefully. In addition to detailed investigations of bumblebee range shifts by scientists, it is important to continue the development of citizen science projects to monitor bumblebee distributions and abundances. Such citizen science projects will also allow us to evaluate how these bumblebees are responding to ongoing climate change over long time periods.

## Methods

### Bumblebee occurrence data

We collected current occurrence data for 15 bumblebee species using photographs taken by citizens in Japan from 2013 to 2015^25^. We focused on six major bumblebee species *B. diversus*, *B. ardens*, *B. hypocrita*, *B. ignitus*, *B. honshuensis*, and *B. beaticola* because the number of photographs of these species was relatively high. *Bombus diversus* and *B. ardens* can be found in various habitats ranging from residential to natural areas, and among Japanese bumblebees, *B. ardens* seem to be most adapted to residential areas. *Bombus ardens* emerge in March, earlier than other Japanese bumblebees, and their colonies collapse in July, earlier than those of other Japanese bumblebees. *Bombus. hypocrita* and *B. honshuensis* are observed in relatively high–altitude regions (the latter tends to live at higher altitudes than the former). *Bombus ignitus* is often observed in low-altitude regions, and is categorized as near threatened by the Ministry of the Environment. *Bombus beaticola* is an alpine species in Japan.

The abovementioned species are relatively easy for experts to identify species from photographic images (see https://hanamaruproject.s1009.xrea.com/hanamaru_project/identification_E.html for the details of their body color and shape). The consistency of species identification for the six studied species by Yokoyama^25^ was 97.7% in our test using 100 bumblebee photographs in which species had been identified two or three years previously.

We used records of *B. diversus* (Fig. 1b), *B. ardens* (Fig. 1c), *B. hypocrita* (Fig. 1d), *B. ignitus* (Fig. 1e), *B. honshuensis* (Fig. 1f), and *B. beaticola* (Fig. 1g) to estimate their distributions. After duplicate records within each cell (1 km x 1 km) were removed, there were 563 points for *B. diversus*, 397 points for *B. ardens*. 251 points for *B. hypocrita*, 192 points for *B. ignitus*, 139 points for *B. honshuensis*, and 70 points for *B. beaticola*. For further details, please refer to Suzuki-Ohno et al.^25^.

### Environmental data

We obtained daily climate data at 1 km resolution for 1978 to 2013 from the National Institute for Agro-Environmental Sciences (NIAES)^56^. The climate variables used in our analyses were total precipitation, mean temperature, and mean solar radiation (Table 1). To minimize the effect of annual fluctuation, we calculated the means of each these three variables for 10 years as past and present climate variables (1978–1987 and 2004–2013). The coefficients of correlation between these climate variables were less than 0.6 (Table S3 in Appendix S3 in Supplementary information). We identified differences between past and present climate variables in each cell. On average, total precipitation increased by 190 mm, mean temperature increased by 0.89 °C, and mean solar radiation decreased by 0.055 MJ m^−2^ in 2004–2013 from 1978–1987.

In general, climate data used to estimate a species distribution are averaged over 30 years to reduce the effect of annual fluctuation on the results. To focused on the effects of land cover change on possible bumblebee range shifts, we used the average climate data at 1 km resolution for a 30-year period (1981–2010). The averaged climate data were obtained from the National Land Numerical Information (NLNI). We selected common variables (annual total precipitation, mean temperature, and mean solar radiation) from the averaged climate data. The coefficients of correlation between these selected climate variables were less than 0.6 (Table S3 in Appendix S3 in Supplementary information).

We obtained land cover data at 1 km resolution for 1987 and 2014 from the NLNI. The NLNI provides land cover data for 2014 based on digital maps of the Geospatial Information Authority of Japan and satellite images of SPOT and RapidEye, and land cover data for 1987 based on topographic maps at a 1:25,000 scale of Geospatial Information Authority of Japan. Golf courses were classified separately in 2014, but included in the “other land” category in 1987. Therefore, we calculated the sum of golf courses and other land areas in 2014, and classified them as “other land areas” (Table 1). The land cover categories used for the estimates were “paddy field area”, “other agricultural area”, “forest area”, “wasteland area”, “other land area”, “building area”, “lake and river area”, “beach area”, and “sea area” (m^2^/km^2^) (Table 1). Please see Appendix S3 in Supplementary information for the details of climate and land cover data.

### Species distribution model, Maxent

Maxent is suitable for estimating species distributions based on the data collected through citizen science monitoring because it requires only presence-only data and environmental data. We used Maxent software version 3.3.3 k to estimate bumblebee distributions. We set the number of replicates as 10, the replicated run type as cross-validate, and the maximum number of background points as 10,000. To reduce sampling effort biases and avoid data overfitting, we performed background manipulation using a bias file based on all occurrence data for 15 bumblebee species (Fig. 1a) following a tutorial^57^, and selected linear and quadratic features (model functions) instead of auto features^58^. In trial experiments, we tested the effect of the regularization multiplier because it avoids data overfitting and enlarges estimated distribution areas^59^. However, increases in the regularization multiplier did not always enlarge estimated distribution areas for six studied bumble bees (Table S4). We considered that data overfitting was sufficiently suppressed by background manipulation and using simple functions, and used default regularization multiplier of 1. Please see Appendix S4 in Supplementary information for more details of the Maxent settings and parameters used in the present study.

In Maxent, the percent contribution represents the relative contribution of each environmental variable to the Maxent estimate, and the permutation importance represents the relative effect of permuting each environmental variable (Table S1 in Appendix S1 and Table S2 in Appendix S2 in Supplementary information). The response curve represents how each environmental variable affects the probability of distribution (Fig. S1 in Appendix S1 and Fig. S3 in Appendix S2 in Supplementary information). Based on these values, we were able to consider which environmental factors primarily affected estimated range shifts.

The AUC represents the accuracy of the Maxent estimate. An AUC value over 0.7 is often considered to be a passing mark (e.g., 0.90–1.00 = excellent; 0.80–0.90 = good; 0.70–0.80 = fair^20^); however, AUC by itself can be a misleading measure of predictive performance^60^. Therefore, we also evaluated the accuracy of estimated past bumblebee distributions using specimen data.

### Evaluation of the accuracy of estimated past bumblebee distributions using specimen data

To verify estimated past bumblebee distributions, we used bumblebee specimen data from the S-Net data portal (https://science-net.kahaku.go.jp) as past distribution test data. The bumblebee specimen data from S-Net primarily included bumblebee data in the Global Biodiversity Information Facility (GBIF; https://www.gbif.jp/v2/). These data were not used as current occurrence data because most of them were old specimen data. In 2017, we extracted data for bumblebee specimens that were collected in the 1980s. Comparison of latitude–longitude data with the specimen collection location showed that the latitude–longitude data were sometimes inaccurate (errors were from several to tens of km). In such cases, we investigated latitude–longitude data using the address of the collection location with Google Maps (https://www.google.com/maps). After removing duplicate records within each cell (1 km $\times$ 1 km), there were 60 points for *B. diversus*, 29 points for *B. ardens*, 39 points for *B. hypocrita*, 17 points for *B. ignitus*, 24 points for *B. honshuensis*, and 14 points for *B. beaticola*. We calculated the percentages of these data points included within the estimated past distributions (i.e., sensitivity). To determine the distribution area based on the probability of distribution, we used the MTP threshold value outputted from Maxent. The MTP is the lowest probability of distribution among training data when a model is estimated using the training data. We also calculated AUC values for estimated past distributions using these data points (Appendices S1 and S2 in Supplementary information).

### Evaluation of possible range shifts

To define estimated bumblebee ranges, we converted the estimated probability of distribution to a binary format (0/1) using thresholds. Among nine thresholds outputted from Maxent, MTP, 10PTP, ETSS, MTSS, BTPT, and EETO are used for converting binary presence–absence data. We used all thresholds (Tables 2, 3). We calculated the number of different cells between the past and present binary presence–absence data. We did not calculate the difference in Hokkaido for *B. ignitus* because this species does not inhabit that island.

### Extraction of land cover change

When the estimated range of a species shrank/expanded due to changes in land cover, we extracted the land cover change and vegetation types for the estimated reduction/expansion areas evaluated using EETO because the EETO is an intermediate threshold between conservative and challenging thresholds and gave the largest estimated reduction areas for *B. diversus*. Land cover change was calculated using land cover data from the NLNI. Vegetation types were extracted from Natural Environmental Information GIS data downloaded from the Biodiversity Center of Japan (https://www.biodic.go.jp).

## Supplementary information

Supplementary Information.
