# Supplementary material for: Estimating possible bumblebee range shifts in response to climate and land cover changes
Source: Sci Rep. 2020 Nov 12;10:19622. doi: 10.1038/s41598-020-76164-5 (PMC7661518; doi:10.1038/s41598-020-76164-5)
Supplement: Supplementary file 1 — Supplementary Information. [file 41598_2020_76164_MOESM1_ESM.docx]

Supplementary information

Estimating possible bumblebee range shifts in response to climate and land cover changes

Yukari Suzuki-Ohno*, Jun Yokoyama, Tohru Nakashizuka, and Masakado Kawata

Appendix S1. Estimating present/past distributions using past/present climate and land cover data (presD-CL and pastD-CL)

In this section, we explain the details of presD-CL [estimated present distributions using present climate data (2004–2013) and land cover data (2014)] and pastD-CL [estimated past distributions using past climate data (1978–1987) and land cover data (1987)]. Area under the curve (AUC), i.e., the area under the receiver operating characteristic (ROC) curve, is generally used as the index of the accuracy of Maxent estimation. The ROC curve represents sensitivity (true positive rate) against fractional predicted area (false positive rate). The mean AUC values of 10 replicates of presD-CL based on current occurrence test data were high for all six species: 0.764 for *Bombus diversus*, 0.820 for *B. ardens*, 0.835 for *B. hypocrita*, 0.890 for *B. ignitus*, 0.932 for *B. honshuensis*, and 0.974 for *B. beaticola*. We also calculated AUC values of pastD-CL based on historical occurrence data (specimen data from the 1980s). The mean AUC values of 10 replicates of pastD-CL based on historical occurrence data were high for two species, but low for four species: 0.635 for *B. diversus*, 0.481 for *B. ardens*, 0.584 for *B. hypocrita*, 0.684 for *B. ignitus*, 0.858 for *B. honshuensis*, and 0.932 for *B. beaticola*. We suggest that these pastD-CL AUC values cannot reflect the accuracy of estimated past distributions due to the strong spatial bias of specimen data (Suzuki-Ohno et al. 2017), especially for common species with wide distributions. The abundances of *B. diversus*, *B. ardens*, and *B. hypocrita* were high in central, northeastern, and northern Japan, and *B. ignitus* was also frequently observed in central and northeastern Japan, but few specimen data were recorded in these areas. Therefore, we determined the mean percentages of historical occurrence data included in the pastD-CLs thresholded by minimum training presence (MTP). The mean percentages were high for all six species: 99.2% for *B. diversus,* 99.7% for *B. ardens*, 99.7% for *B. hypocrita*, 100% for *B. ignitus*, 87.1% for *B. honshuensis*, and 85.7% for *B. beaticola*.

The environmental variables used for these estimations are given in Table 1. Percent contribution represents the relative contribution of each environmental variable to the Maxent estimate, and permutation importance represents the relative effect of permuting each environmental variable. The percent contribution and permutation importance of important environmental variables are shown in Table S1. The marginal response curve represents how an environmental variable changes the Maxent prediction using average sample values for other environmental variables (Fig. S1).

The percent contribution of mean temperature was high for all six species (Table S1). A relatively high temperature is suitable for *B. diversus* and *B. ignitus*, but an excessively high temperature is not suitable for these two species (Fig. S1a and d). A moderate temperature is suitable for *B. hypocrita* and *B. honshuensis*, and a low temperature is suitable for *B. beaticola* (Fig. S1c, e and f). Over the past 26 years, mean temperatures have increased in almost all regions of Japan, and the mean temperature in 2004–2013 was on average 0.89 ºC higher than that in 1978–1987 in Japan. Therefore, the estimated ranges of these five species was reduced by global warming. In contrast, a higher temperature is suitable for *B. ardens* (Fig. S1b), and the range of this species was estimated to expand due to global warming. The percent contribution of mean solar radiation was also high for five species (Table S1). Although the percent contribution of mean solar radiation was higher than that of mean temperature for *B. diversus* and *B. ignitus* (Table S1), changes in solar radiation differed by regions and did not correspond closely to the areas of estimated *B. diversus* and *B. ignitus* range reduction. The percent contribution of forest area was also high for four species, but large changes in forest area occurred in small local regions.

The locations of estimated range reductions depended on thresholds. However, the estimated *B. beaticola* range reductions were observed on the northern island Hokkaido regardless of thresholds (Fig. S2).

Table S1. The effects of environmental factors on presD-CL [estimated present distributions using present climate data (2004–2013) and land cover data (2014)]. These results were the mean values of 10 replicates. Upper numbers are percent contribution, and bottom numbers are permutation importance. We omitted the environmental factors with a percent contribution of less than 5% for all six species. Prec: annual precipitation, Temp: annual mean temperature, Solar: mean solar radiation, FR: forest, OL: other land (artificial open space with neither woods nor buildings), BL: building.

|  | Prec | Temp | Solar | FR | OL | BL |
| --- | --- | --- | --- | --- | --- | --- |
| *B. diversus* | 3.5 | 23.3 | 25.6 | 30.2 | 9.8 | 1.2 |
|  | 6.7 | 23.9 | 28.1 | 22.8 | 3.4 | 13.7 |
| *B. ardens* | 5.2 | 23.3 | 4.3 | 13.7 | 1.7 | 45.3 |
|  | 4.1 | 22.9 | 11.5 | 13.2 | 1.2 | 42.8 |
| *B. hypocrita* | 0.5 | 33.9 | 31.0 | 14.8 | 7.7 | 3.7 |
|  | 1.0 | 47.5 | 26.2 | 13.4 | 2.5 | 5.8 |
| *B. ignitus* | 9.3 | 18.0 | 23.0 | 39.6 | 1.2 | 0.8 |
|  | 3.1 | 40.4 | 16.1 | 9.9 | 2.7 | 17.7 |
| *B. honshuensis* | 2.4 | 39.8 | 34.4 | 6.9 | 9.8 | 1.2 |
|  | 1.3 | 26.2 | 6.2 | 33.9 | 7.2 | 7.8 |
| *B. beaticola* | 0.6 | 60.9 | 26.7 | 0.2 | 7.9 | 2.4 |
|  | 0.8 | 86.0 | 5.3 | 1.1 | 1.6 | 4.9 |


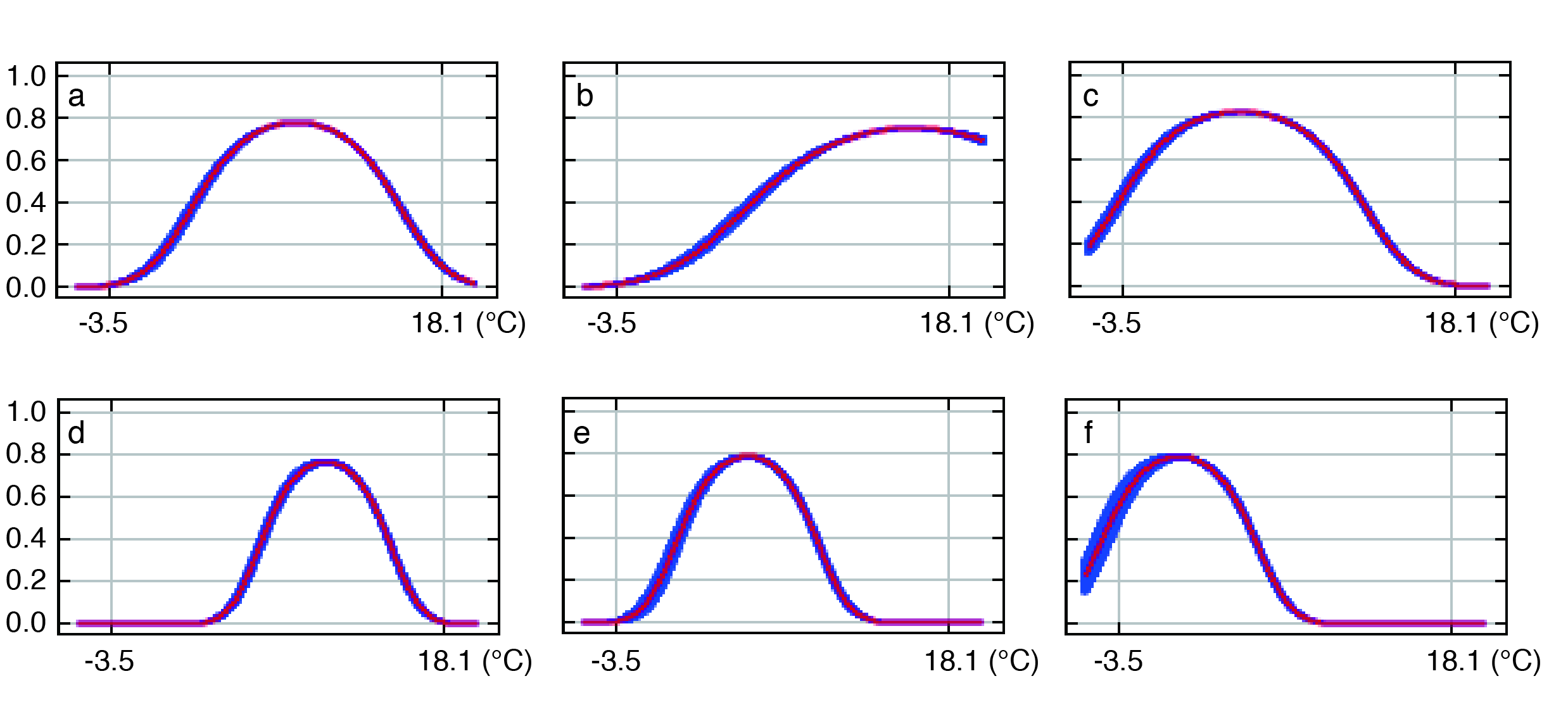


Figure S1. Marginal response curves to temperature for presD-CL [estimates using present climate data (2004–2013) and present land cover data (2014)]. (a) *Bombus diversus*; (b) *B. ardens*; (c) *B. hypocrita*; (d) *B. ignitus*; (e) *B. honshuensis*; and (f) *B. beaticola*.


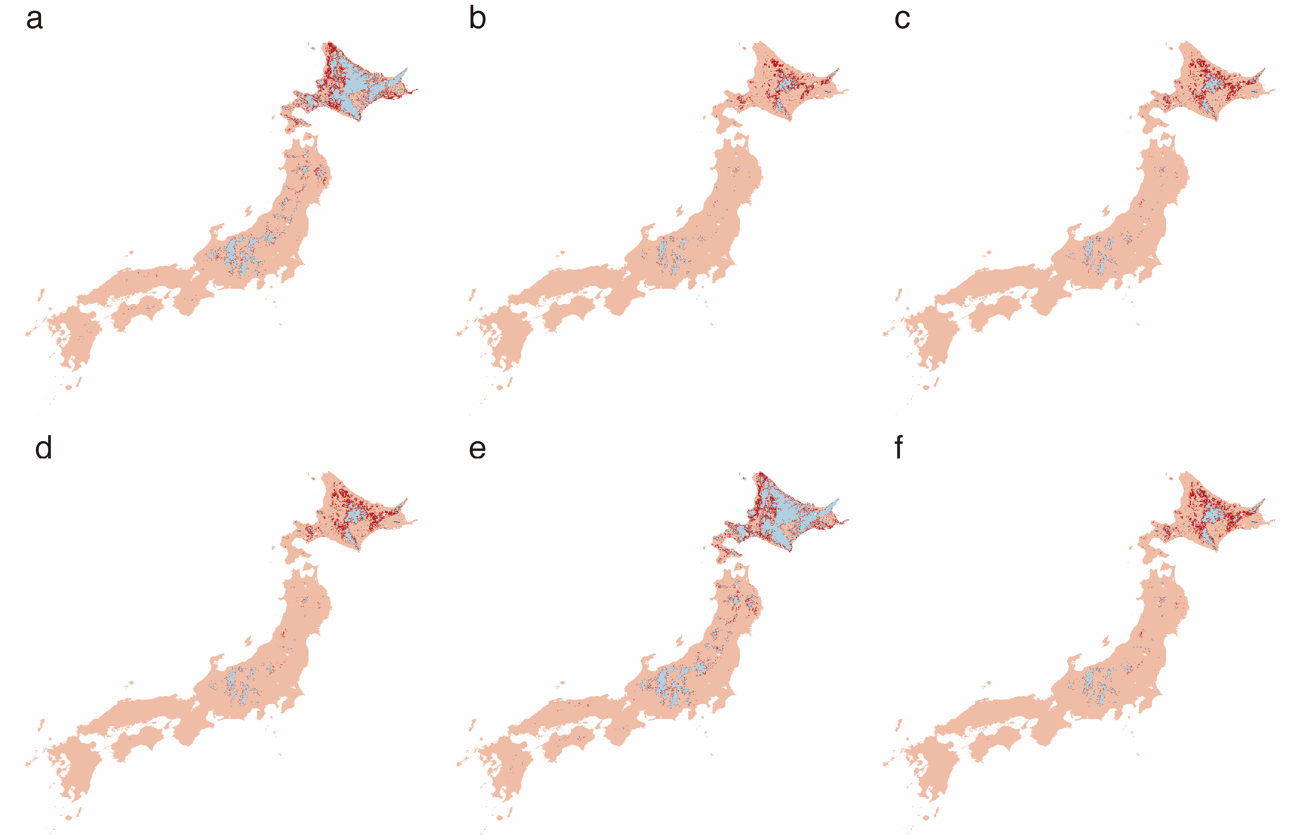


Figure S2. Possible range shifts for *Bombus beaticola* evaluated based on different thresholds. (a) MTP; (b) 10PTP; (c) ETSS; (d) MTSS; (e) BTPT; and (f) EETO. Blue and red represent estimated range reduction and expansion, respectively. Sky blue indicates no difference in estimated present areas, whereas pink indicates no difference in estimated absent areas. This map was drawn with the software QGIS ver. 2.14 (https://qgis.org/en/site/).

Appendix S2. Estimating present/past distributions using average climate data and past land cover data (presD-L and pastD-L)

In this section, we explain the details of presD-L [estimated distributions using averages of 30 years of climate data (1981–2010) and present land cover data (2014)] and pastD-L [estimated past distributions using averages of 30 years of climate data (1981–2010) and past land cover data (1987)]. The mean AUC values of 10 replicates of presD-L based on current occurrence test data were high for all six species: 0.756 for *B. diversus*, 0.817 for *B. ardens*, 0.822 for *B. hypocrita*, 0.883 for *B. ignitus*, 0.936 for *B. honshuensis*, and 0.973 for *B. beaticola*. We also calculated the AUC values of pastD-L based on historical occurrence data (specimens). The mean AUC values of 10 replicates of pastD-L based on historical occurrence data were high for two species, but low for four species: 0.605 for *B. diversus*, 0.518 for *B. ardens*, 0.574 for *B. hypocrita*, 0.634 for *B. ignitus*, 0.863 for *B. honshuensis*, and 0.943 for *B. beaticola*. We suggest that these pastD-L AUC values cannot reflect the accuracy of estimated past distributions due to the large spatial bias of specimen data (Suzuki-Ohno et al. 2017), especially for common species with wide distributions. The abundances of *B. diversus*, *B. ardens*, *B. hypocrita* were high in central, northeastern, and northern Japan, and *B. ignitus* was frequently observed in central and northeastern Japan, but few specimen data were recorded in these areas. Therefore, we determined mean percentages of historical occurrence data included in the pastD-Ls thresholded by minimum training presence (MTP). The mean percentages were high for all six species: 98.2% for *B. diversus,* 99.7% for *B. ardens*, 99.0% for *B. hypocrita*, 100% for *B. ignitus*, 83.3% for *B. honshuensis*, and 78.6% for *B. beaticola*.

The percent contribution of forest area was high for *B. diversus*, *B. ardens*, *B. hypocrita*, and *B. ignitus* (>14% in Table S2). There are specific forest areas that are suitable for these four species (Fig. S3). For *B. ignitus*, the marginal response curve seems to increase as forest area increases (Fig. S3d). However, when a species distribution model was created using only forest area, the response curve of forest area peaked at 35% of 1 km^2^ (data not shown). Increase in forest area would cause estimated range reductions for *B. diversus,* *B. hypocrita*, and *B. ignitus* (Fig. 4). The major land cover changes in the areas of estimated range reduction evaluated based on EETO for *B. diversus,* *B. hypocrita*, and *B. ignitus* was an increase in forest area (Fig. 4a, b, and c), and one of the major land cover changes in the areas of estimated range expansion was a decrease in forest area (Fig. 4d, e, and f). On the other hand, a decrease in forest area was not frequently observed in the areas of estimated *B. ardens* range expansion (Fig. S4d). The percent contribution of building area was much higher than that of forest area (Table S2), and an increase in building area was observed in the areas of estimated *B. ardens* range expansion (Fig. S4d). A decrease in OL area (artificial open space with neither woods nor buildings) was observed in the areas of estimated *B. honshuensis* and *B. beaticola* range reduction evaluated based on EETO (Fig. S4b and c), and an increase in OL area was observed in the areas of estimated *B. honshuensis* and *B. beaticola* range expansion (Fig. S4e and f). The percent contribution of OL for *B. honshuensis* and *B. beaticola* was the highest among those of land cover types (Table S2). As *B. honshuensis* and *B. beaticola* inhabit high-altitude regions, a suitable OL for *B. honshuensis* and *B. beaticola* might correspond to grasslands, including parks and ski areas managed by humans.

Table S2. The effects of environmental factors on presD-L [estimated present distributions using average climate data (1981–2010) and present land cover data (2014)]. These results were the mean values of 10 replicates. Upper numbers are percent contribution, and bottom numbers are permutation importance. We omitted the environmental factors with a percent contribution of less than 5% for all six species. Prec: annual precipitation, Temp: annual mean temperature, Solar: mean solar radiation, FR: forest, OA: other agricultural land (agricultural land other than paddy field), OL: other land (artificial open space with neither woods nor buildings), BL: building.

|  | Prec | Temp | Solar | FR | OA | OL | BL |
| --- | --- | --- | --- | --- | --- | --- | --- |
| *B. diversus* | 0.9 | 23.1 | 24.1 | 33.9 | 4.0 | 9.7 | 1.4 |
|  | 2.1 | 22.0 | 26.6 | 25.7 | 0.8 | 2.1 | 18.1 |
| *B. ardens* | 4.3 | 24.7 | 3.4 | 14.1 | 1.3 | 2.5 | 44.2 |
|  | 5.4 | 32.8 | 10.2 | 21.2 | 1.2 | 0.8 | 19.4 |
| *B. hypocrita* | 2.0 | 33.1 | 25.7 | 18.3 | 2.6 | 8.8 | 4.1 |
|  | 0.3 | 39.6 | 22 | 12.9 | 3.3 | 3.7 | 14.6 |
| *B. ignitus* | 14.5 | 19.4 | 7.8 | 43.1 | 6.0 | 1.1 | 2.6 |
|  | 6.8 | 17.7 | 0.5 | 15.0 | 14.3 | 5.2 | 23.2 |
| *B. honshuensis* | 1.4 | 39.8 | 34.6 | 7.3 | 1.0 | 9.8 | 1.8 |
|  | 0.4 | 22.6 | 1.8 | 34.3 | 10.3 | 8.9 | 8.9 |
| *B. beaticola* | 0 | 62.6 | 24.7 | 0.3 | 1.2 | 8.8 | 2.3 |
|  | 0 | 87.7 | 5.7 | 1.1 | 0 | 2.0 | 3.4 |


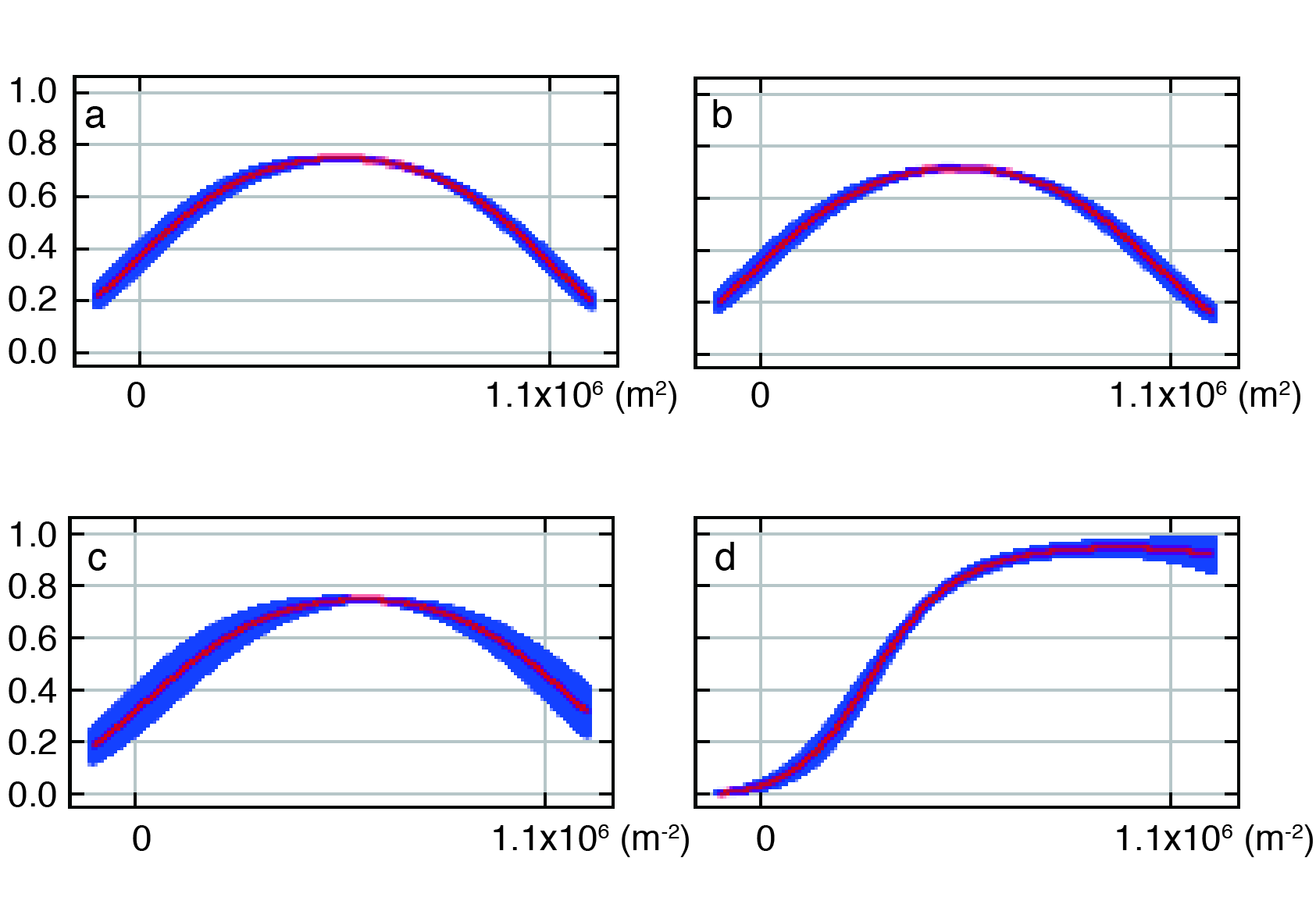


Fig. S3. Marginal response curves to forest area for presD-L [estimates using average climate data (1981–2010) and present land cover data (2014)]. (a) *Bombus diversus*, (b) *B. ardens*, (c) *B. hypocrita*, and (d) *B. ignitus*.





Fig. S4. Turkey boxplot of the changes of land cover areas in areas of range reduction and expansion evaluated based on EETO. EETO is equate entropy of thresholded and original distribution. These are the boxplot of the mean change areas in each estimate for 10 estimates by Maxent. Upper and lower graphs are the mean change areas in the areas of range reduction and range expansion, respectively. (a), (d) *Bombus ardens*; (b), (e) *B. honshuensis*; (c), (f) *B. beaticola*; FR: forest, PD: paddy field, OA: other agricultural land (agricultural land other than paddy fields), WL: wasteland, OL: other land (artificial open space with neither woods nor buildings), BL: building.

Appendix S3. Environmental data

We used climate and land cover data at 1 km resolution for the Japanese archipelago, excluding the Kurile Islands and the Ryukyu Islands south of Yakushima Island (the southern limit of bumblebee distribution in Japan). We used 12 variables (Table 1) to estimate bumblebee distributions.

*Climate data*

Daily climate data from 1978 to 2013 were obtained from the National Institute for Agro-Environmental Sciences (NIAES) (Seino 1993). These climate data at 1 km resolution were estimated using data from the Automated Meteorological Data Acquisition System (AMeDAS), which exists at about 1,300 sites in Japan. The summary of the daily climate values every year lists 117 variables, including annual total precipitation, annual mean temperature, annual mean solar radiation, annual mean relative humidity, annual mean wind speed, total precipitation each month, mean/max./min. temperature each month, mean solar radiation each month, mean relative humidity each month, mean wind speed each month. Since a strong correlation between environmental variables causes misleading Maxent estimations, we selected annual total or annual mean values because the max/min. values are often strongly correlated with mean value. We did not select relative humidity or wind speed variables because they are not listed in the average climate data for a 30-year period. Therefore, we selected annual total precipitation, annual mean temperature, and annual mean solar radiation as the climate data used for estimates in Maxent. Precipitation, temperature, and solar radiation are important climate variables for bumblebees (Bergman et al. 1996, Woodard et al. 2017, Ogilvie et al. 2017, Soroye et al. 2020). To reduce annual fluctuation, we calculated the mean of these three variables for 10 years as past and present climate variables (1978–1987 and 2004–2013). We calculated correlations between the mean variables using Perl. The correlation coefficients between the mean variables were less than 0.6 (Table S3). We converted the list of these climate variables with mesh ID (i.e., locations) into a csv file to raster data for each variable using ArcGIS 10.0.

In general, climate data used to estimate species distribution are averaged over 30 years to reduce the impact of annual fluctuation on the results. Therefore, to see the effects of land cover change alone on bumblebee range shifts, we used the average climate data for a 30-year period (1981–2010). The averaged climate data were obtained from the National Land Numerical Information (NLNI). The environmental data listed 83 variables, including total precipitation, annual mean temperature, annual mean solar radiation, total precipitation each month, mean/max./min. of temperature each month. We selected common variables (annual total precipitation, annual mean temperature, and annual mean solar radiation) from NIAES. The correlation coefficients between them were less than 0.6 (Table S3). The averaged climate data were recorded in a shape file and converted into raster data for each climate variable using ArcGIS 10.0.

*Land cover data*

The land cover data for 1976, 1987, 1991, 1997, 2006, 2009, and 2014 can be obtained from the NLNI (http://nlftp.mlit.go.jp/ksj/index.html). We used the land cover data for 2014 as present data, and those for 1987 as past data. We did not use land cover data for 1976 because these data included erroneous values and characters instead of numbers. Land cover data for 2014 was based on digital maps from the Geospatial Information Authority of Japan and SPOT and RapidEye satellite images, whereas land cover data for 1987 was based on topographic maps at a 1:25,000 scale from the Geospatial Information Authority of Japan.

The areas covered by paddy fields, other agricultural land, forest, wasteland, building, other land (i.e., artificial open space with neither woods nor buildings), golf courses, roads, railroads, rivers and lakes, beaches, and sea (m^2^/km^2^) within a 1 km cell were listed in the land cover data. Road and railroad areas were not used for Maxent estimates because these areas were small and they contributed only a small percentage to the model (<1%). As golf courses were included in the “other land” category in 1987, we calculated the sum of golf courses and other land areas in 2014 (Table 1). In addition, past land cover data included some erroneous values (e.g., 9999999) for islands and coastlines. We excluded these erroneous values in the 1987 data from the analysis of bumblebee range shifts.

Table S3. Correlation coefficients of climate variables.

| Variables (year) | Coefficient |
| --- | --- |
| Precipitation and temperature (2004–2013) | 0.328 |
| Precipitation and solar radiation (2004–2013) | 0.265 |
| Temperature and solar radiation (2004–2013) | 0.577 |
| Precipitation and temperature (1978–1987) | 0.384 |
| Precipitation and solar radiation (1978–1987) | 0.207 |
| Temperature and solar radiation (1978–1987) | 0.404 |
| Precipitation and temperature (1981–2010) | 0.332 |
| Precipitation and solar radiation (1981–2010) | 0.294 |
| Temperature and solar radiation (1981–2010) | 0.451 |

Appendix S4. Maxent settings and parameters

In studies that estimate species distributions with Maxent, the spatial bias of sampling efforts in citizen science monitoring sometimes becomes a problem. To reduce sampling effort bias in citizen science monitoring, we performed background manipulation with a bias file that limits background areas according to the sampling effort bias (Young et al. 2011). We made a bias file based on the municipalities map provided by ESRI and the occurrence data of all bumblebee species (Fig. 1a). The bias file was specified in the Advanced panel in Maximum Entropy Parameters. Spatial filtering is also effective in reducing sampling effort bias, but we did no spatial filtering due to the small volume of occurrence data (but duplicate records were removed in the Basic panel in Maximum Entropy Parameters). In studies estimating species distributions with Maxent, auto features sometimes cause problems because they create complex functions that lead to data overfitting. To avoid data overfitting, we selected linear and quadratic features instead of auto features (Merow et al. 2013; Syfert et al. 2013, 2014).

In trial experiments, we tested the effect of a regularization multiplier because it avoids data overfitting and enlarges estimated distribution areas (Radosavljevic & Anderson 2014). However, increases in the regularization multiplier did not always enlarge estimated distribution areas for bumblebees (Table S4). The proportion of range reduction/expansion was not dramatically changed in most cases. We suggest that data overfitting was sufficiently suppressed by using background manipulation and simple functions (i.e., linear and quadratic features). Therefore, we used default regularization multiplier of 1.

Table S4. Examples of the effects of a regularization multiplier (β = 1, 2, and 4) on the estimated distribution areas and range shifts using present/past climate and land cover data. Upper values are the estimated present distribution areas and bottom values are the percentage of estimated range reduction/expansion. The distribution areas and range reduction/expansion were evaluated by maximum training sensitivity plus specificity.

|  | β = 1 | β = 2 | β = 4 |
| --- | --- | --- | --- |
| *B. diversus* | 95652.7  -13.9% | 89916.4  -14.2% | 95493.7  -14.5% |
| *B. ardens* | 98874.2  17.3% | 100576.7  17.4% | 94186.3  21.3% |
| *B. hypocrita* | 62476.8  -30.7% | 65908.5  -31.0% | 75838.9  -38.1% |
| *B. ignitus* | 90583.2  -27.6% | 95470.5  -26.5% | 87442.6  -24.5% |
| *B. honshuensis* | 28373.4  -19.7% | 25091.1  -17.2% | 27536.7  -15.1% |
| *B. beaticola* | 36623.1  -48.8% | 31460.9  -50.9% | 35997.1  -52.1% |

References

Kinota, K., Takamizawa, K., & Ito, M. The Bumblebees of Japan. Hokkaido university press (2013).

Merow, C., Smith, M.J., & Silander Jr., J.A. A practical guide to MaxEnt for modeling species’ distributions: what it does, and why inputs and settings matter. *Ecography* **36**, 1058-1069 (2013).

Radosavljevic, A. & Anderson, R.P. Making better Maxent models of species distributions: complexity, overfitting and evaluation. *Journal of biogeography* **41**, 629-643 (2014).

Seino, H. An estimation of distribution of meteorological elements using GIS and AMeDAS data. *Journal of Agricultural Meteorology* **48**, 379-383 (in Japanese) (1993).

Soroye, P., Newbold, T., & Kerr, J. Climate change contributes to widespread declines among bumble bees across continents. *Science* **367**, 685-688 (2020)

Woodard, S.H. Bumble bee ecophysiology: Integrating the changing environment and the organism. Curr. Opin. Insect Sci. **22**, 101-108 (2017).

Ogilvie, J.E. et al. Interannual bumble bee abundance is driven by indirect climate effects on floral resource phenology. *Ecology Letters* **20**, 1507-1515 (2017).

Suzuki-Ohno, Y., Yokoyama, J., Nakashizuka, T., & Kawata, M. Utilization of photographs taken by citizens for estimating bumblebee distributions. *Scientific Reports* **7**, 11215. doi: 10.1038/s41598-017-10581-x (2017)

Syfert, M.M., Smith, M. J., & Coomes, D.A. The effects of sampling bias and model complexity on the predictive performance of MaxEnt species distribution models. *PLoS One* **8**, e55158. doi: 10.1371/journal.pone.0055158 (2013).

Syfert, M.M., Joppa, L., Smith, M.J., Coomes, D.A., Steven, P.B., & Brummitt, N.A. Using species distribution models to inform IUCN Red List assessments. *Biological Conservation* **177**, 174-184 (2014).

Young, N., Carter, L. & Evangelista, P.A. MaxEnt Model v3.3.3e Tutorial (ArcGIS v10). http://ibis.colostate.edu/WebContent/WS/ColoradoView/TutorialsDownloads/A_Maxent_Model_v7.pdf (2011).
